# Abstract of the Report of the Committee on Foreign Relations of the National Association of Dental Faculties

**Published:** 1898-11

**Authors:** 


### Abstracts and Translations.

### ABSTRACT OF THE REPORT OF THE COMMITTEE ON FOREIGN RELATIONS OF THE NATIONAL ASSOCIATION OF DENTAL FACULTIES.[1]

To the President and Members of the National Association of Dental Faculties:

The special committee on recognition of degrees in foreign countries and the comparative value of foreign degrees in this country, appointed at the last meeting of this Association, begs leave respectfully to report as follows:

The scope of the investigation of the committee has been somewhat changed from that which would appear to be indicated by the announcement in the published proceedings. It should not be forgotten that there are really no foreign degrees in dentistry, the nearest approach to this being the licentiate in England. America is peculiar in having a distinct and separate diploma for the graduates of distinctly dental colleges. We cannot hope for the recognition of this distinction until our course of instruction is fully comprehended in Europe, and the reputability of our degree established. The report of your committee, then, will specially consider the preliminary steps which we believe it proper to take before commencing further agitation. The subject is of the deepest significance, and involves our whole system of education. There can be no mutual recognition until there has been secured some common ground on which the profession of the various countries of Europe and of America can meet. At present the systems are too diverse, and involve too many seeming contradictions to allow any real reciprocity. Yet that is a consummation devoutly to be wished, and certainly we in America should spare no pains in the endeavor to bring it about. Hence the appointment of a committee to take the subject into consideration by the supreme authority in matters educational among us was doubtless a wise movement, and one which, in the opinion of your committee, should be persistently followed up. Indeed, the committee has received many letters highly approving of the action taken, with the promise of a hearty cooperation on the part of American dentists resident abroad.

---

[1] Made at Omaha, Nebraska, August 29, 1898.

That this body may act intelligently, it seems necessary in this report to review, as concisely as is possible with thoroughness, the real situation, with the view of securing a better state of affairs.

It must be remembered that scholastic dental practice, with the separate teaching which has been found necessary, is of quite recent origin. The first distinctive dental school was established in America in 1840, less than sixty years ago. For more than forty years all didactic and class instruction was confined to this country. Within the past twenty years dental schools have been organized in some of the countries of Europe, usually in connection with hospitals, for the purpose of securing clinical instruction and material. There has never been any reciprocity between the schools of the two continents, if we except the recognition temporarily accorded by England to the dental departments of Harvard and Michigan Universities. The conditions obtaining in the two continents too widely vary. The one is long settled, possessing the real erudition that can only be found in nations that have a past, but imperatively dominated by the traditions and precedents which are the natural outgrowth of heredity. The other has been a new country, with no educational or other institutions hallowed by centuries of growth and progress, and possessing the weight of ancestral influence. In the settlement and development of this country our people encountered obstacles totally unknown to the older states of Europe. Precedent there was none to guide, and tradition there was none to influence. The problems which confronted them were conditions existing, and not theories for consideration. The forces of nature were in one sense our foes, and not our allies. It was necessary first to overturn and reconstruct that which nature had already constructed. The struggle to accomplish this made of us a practical, inventive, ingenious people, who care mainly for ends and little for methods, while Europe respects no practical accomplishment that is secured through irregular, unacknowledged methods.

All these matters are reflected in the status of the dental profession here and abroad. Europe cannot be brought to believe in a practice not founded on a liberal preliminary education, while we are, as a whole, too careless concerning antecedents, so long as anything practical is assured. In Europe, if a man has a university education he is popularly supposed to be competent to practise any profession,—law, medicine, divinity, or any of the specialties,—any distinct instruction being required only to make him acquainted with the tools that he must use. The university degree is supposed to include everything lesser, and hence there is no necessity for any other, that comprising all.

We in America have instinctively recognized the desirability of a university training by founding many schools without sufficient endowment for their independent support, thus really cheapening the university course. This has been through the endeavor to extend educational facilities to the masses, here again looking towards ends and not means.

The natural consequence of all this to our profession has been that in America to-day are probably found the best-skilled operators and the highest development of practical work, while in erudition we are in the rear of several European countries.

It may thus be seen that it is difficult to find a common ground on which the professions of the Old and the New World can stand in equality. Europe will tolerate nothing that does not bear the stamp of regularity. We are satisfied with anything that accomplishes the end sought.

The curricula of the schools of the two continents materially differ. But that could be overlooked, or they might be harmonized. The essential variation lies in the methods or way through which admission within the ranks of the profession is secured. The old countries jealously guard the doors of entrance. We throw them wide open, or, at the best, erect a barrier that is too easily overleaped. Europe declares that the learned professions must be reserved for the learned classes, and that any who enter must come through the door of a liberal education. We urge that the only sufficient qualification is fitness and practical knowledge. Europe will never come to our stand-point. Can we, or should we, attempt to reach hers? In the process of time this may undoubtedly be brought about. Already in America we see the effect of a comparatively low standard of requirements in the overcrowding of the professional ranks, so that they are losing their distinctive respectability and status, and with that their influence for good is circumscribed. Should this acceptation of the professional tone continue for any length of time, there will be no distinction between the professions and trade. Indeed, we find to-day many who contend that no line of demarcation should be drawn. Colleges of law, medicine, and divinity, with their specialties of dentistry, pharmacy, etc., are so multiplying that the consequence must eventually be self-destruction and the annihilation of all professional sentiment. A limit must be placed on the number of schools, and this can only be done by raising the professional standard to a point that will shut out unworthy and unqualified colleges and their students. When this is done, and our preliminary educational standard is sufficiently advanced, with our practical methods and operative skill we shall be prepared to force the profession of Europe to come to our standard, if we are in advance of them, while if they are ahead of us we will be equally bound to reach their level. Even as it is, we are fast approaching each other, they growing more practical and we more erudite. To hasten the desirable end, in the opinion of your committee, this Association should endeavor to secure the co-operation of our *confrères* of the different countries by some official reciprocity, and we believe that the best method to accomplish this would be through the appointment of a standing committee on foreign relations, whose duty it shall be to make us better acquainted with European educational methods and curricula and to inform them of the advantages of ours. This might smooth many of the asperities and remove many of the prejudices which work to the detriment of both at the present time.

The second matter referred to this committee involves our relations with our American *confrères* living and practising abroad. This is closely allied to the subject already considered. American dentists practising in Europe have bitterly complained of the granting of the peculiar American degree to those who are in foreign countries considered unqualified. There is no questioning the fact that this has been done in the past. The time once was when foreigners flocked to our shores to complete their dental education by an American course of study, and by the securing of an American degree. This was materially checked by irresponsible institutions which conferred their honors too indiscriminately. The American degree was fast falling into such disrepute that it became necessary to do something, and accordingly this national organization of teachers was formed. I need not enlarge upon its great accomplishments. But unfortunately it was not conceived soon enough. Cause for reproach had already been given, and Europe has not hesitated to take advantage of it to her own benefit and in her own interests, and hence the D.D.S. does not now receive the consideration to which of right it is entitled, nor has sufficient credit been accorded to the work of this Association. It takes a long time to live down the bad reputation that may be gained in a day.

Two things are charged by American dentists practising in Europe,—

First, That students from the old countries are received by our schools and given advanced standing on the presentation of certificates in foreign tongues which are really worthy no consideration whatever.

Second, That diplomas are practically sold by American institutions and degrees conferred *in absentia.*

There is, unfortunately, no disputing the fact that our *confrères* abroad have sometimes had cause for complaint that the value of their diplomas has been depreciated, and that they have not been sufficiently protected by the schools granting them. Even since the organization of this association of colleges its rules governing the admission of students have been violated on different occasions, through ignorance of the value of some of the certificates which the regulations have made necessary for advanced standing. It is also more than probable that worthless and even fraudulent certificates have sometimes been used as pretexts for giving advanced standing in certain American colleges, when their real character should have been well known by the authorities. A foreigner who desires an American degree, and who occupies but a low social position at home, procures a certificate from some unqualified source, perhaps under false representations. It is written in a foreign tongue and sealed with some pretentious seal, possibly that of an emigration or other bureau. This he presents to the American college, assuring the authorities that it represents a definite course of dental study. The dean is perhaps unable or indisposed to have it verified, and it is accepted, the applicant under it is admitted to the senior course, and graduated at the end of a single term. Thus, after an absence of but a few months, the student, perhaps a servant or a barber's apprentice who had become possessed of a little money, returns to his native land and flourishes in the faces of his former associates a diploma that should be the distinguishing characteristic of an educated man, and claims to be the *confrère* of those who have honestly earned a certificate of fitness from an American school, and upon whose diploma this unmerited scandal and disgrace has thus been brought.

It is possible that the institution thus offending may have been nothing more than careless. It would take weeks to verify the certificate presented, and then it would be too late for entrance. There are no means at hand by which the value of the document can be ascertained, and so the applicant is given the benefit of the doubt and admitted to advanced standing.

Formal complaint was last year made by American dentists in Switzerland in the case of a student who was admitted to the

Senior class of a college having membership in this body. He had been permitted to join upon the presentation of a foreign certificate. Culpable negligence seemed to have been exercised, and had it not been for the energetic protest of our *confrères* abroad the student would have been graduated at the end of a few months. Upon the presentation of the case the college reduced the student to the Freshman class, and he must wait for his diploma. It is further charged that he was matriculated when not in this country, the date of the closing of the time for registration having expired before his arrival in America. Such cases as this should be closely investigated, that the offending college may be punished if guilty, or exonerated if innocent.

It has appeared impossible in many instances to determine the character of the certificates presented. The foreign school, or pretended school, is unknown here. We have no list of such, and great injustice might be done to applicants if the document is refused, provided it be genuine and sufficient. But it is quite proper for every college to insist upon the endorsement of some known authority. If, as it now is, the authorities are conscientious in the matter and ask for a verification of the document presented, the prospective student perhaps brings a countryman who is suborned to give a false interpretation of it. Or he goes to a rival school, representing that the first to which he applied, and which calls for the additional testimony, had accepted him, but that he had found the college to be inferior to its neighbor, and so he wishes to transfer his matriculation to a better one. That appeals to more than one perverted sense, and he is accepted on his mere assertion, skilfully made, that the document had been approved by the other institution, and is given advanced standing.

As for the determination of preliminary qualifications, that is a yet more difficult affair. The systems of general education in different countries are so diverse that it is almost an impossibility to decide what may be accepted as the equivalent for the standard of the National Association of Dental Faculties. And so the reception of students from abroad is a matter in which the most conscientious dean may be at fault.

This condition of affairs has long existed. It forms the basis for many bitter complaints on the part of both foreign and American dentists practising abroad. It very loudly calls for reform, and to your committee it seems that the good name and reputation of this Association is concerned, and that we are in honor bound to seek some remedy.

The communications from abroad that have been referred to this committee suggest that a board of European dentists should be appointed by this body, who shall take cognizance of such cases, and whose endorsement of the status of a proposed student shall be necessary for his matriculation in any recognized college. To give to such a foreign and irresponsible board plenary powers in the acceptance of applicants for matriculation from abroad is of course quite impossible. We have no legal or moral right to delegate the authority that has been by law vested in the responsible faculties of our colleges. In some of the States the determination of the qualifications is vested in State authorities, and they could not and would not delegate it to any board whatever. In the State of New York the college officers have nothing whatever to do with the determination of the preliminary qualifications of applicants. They must obtain from the State Regents a dental student's certificate before they can be accepted.

But your committee can see no objection to the naming of an advisory board, whose endorsement of any paper and whose certificate of educational and moral status may be considered sufficient, and it therefore recommends that not more than three qualified persons, resident in each of the principal countries of Europe, be appointed as an advisory board, to whom students from abroad may present their certificates of qualification and moral character for endorsement, or to whom the papers of students from abroad concerning which there is doubt or uncertainty may be referred for authentication and approval. Your committee was advised that this matter would be brought before the American Dental Society of Europe at its meeting in London, August 1, and that the chairman would be apprised of any action there taken.[1]

The second cause of complaint that has been urged before your committee is that some institutions with high-sounding titles and names, and which are perhaps endorsed by State officers as having legal status, practically sell their diplomas abroad. This complaint is also one of long standing, and your committee believes that it is well founded. The condition is one for which, however, this Association is not responsible. Yet it seriously reflects upon American educational institutions, and is a source of scandal and

---

[1] Since this report was presented and adopted the chairman of the committee has received an abstract of the proceedings, in which was recommended the very action taken by the National Association of Dental Faculties at its late annual meeting.

opprobrium which cannot be ignored by it. In foreign countries the embarrassments of the situation are not comprehended. Under all the European governments it is possible to enact a general law that shall be effectual. We have nearly fifty separate States, each autonomous so far as its domestic affairs are concerned, and all educational matters belong in that category. Hence one State may enact a law under which it is possible to incorporate an institution essentially fraudulent in its character, and the other States are powerless to prevent or correct the evil.

The State of Illinois is a glaring example of this kind of vicious legislation, and nearly or quite all the fraudulent colleges are now located in the city of Chicago, to the great reproach of the State and the profession of dentistry within its borders. That city contains some of the very best of our professional educational institutions, and at the same time the most villanous impostures conceivable. Dentistry in Chicago can boast of as high-toned and eminent practitioners as are found anywhere in the world, and it is disgraced by some who appear to acknowledge none of the usually accepted professional obligations, while using the professional name to further their own illegitimate ends. Unfortunately it is sometimes hard for the uninitiated to tell them apart, for some of the latter have held responsible professional positions, and use that seeming endorsement in the pursuit of their illicit business.

Men unacquainted with professional educational affairs, who know not the wiles of designing tricksters who would take advantage of an innocent law to further their own selfish purposes, are not the best judges of what is proper legislation for the profession. In an unsuspecting moment, and without sufficient consideration, there was placed upon the Illinois statute-books an enactment which, while assuming to further business interests and honestly intended for their benefit, allows the incorporation under the law of associations that may carry on a fraudulent diploma business. So loosely or so nefariously drawn was this law that for the merely nominal fee of registration, amounting to less than five dollars, totally unqualified men may be permitted to issue diplomas of qualification in the different professions. This seems a monstrous state of affairs, but it has been suffered to exist for years. The citizens of other States are powerless, for Illinois is supreme within her own jurisdiction, and she continues to protect her criminals in their villany. The task of securing the repeal of this vicious law is too great for the courage of its reputable men, for ignorance and vice have struck hands in its maintenance. Even the excellent and influential Illinois State Dental Society has looked upon this condition with seeming indifference. As a consequence of the continuance of this demoralizing law, a considerable number of the practitioners of Chicago carry in their pockets or exhibit on their walls college charters conferring upon them the power to issue diplomas in dentistry. A number of advertising offices are legally conducted under such names as "The Illinois Academy of Medicine and Dentistry," "The College of Painless Dentistry," "The Union College of Dentistry," etc., and, *mirabile dictu*, the certificate of the Secretary of State, under the great seal of the State of Illinois, can be obtained certifying to their entire legal respectability and status. It seems to your committee that the decent part of the profession of this grand State should begin an agitation for the repeal of this vicious law. It is earnestly to be hoped that as soon as the professional men of the State are aroused from their lethargy and made to comprehend the enormity of the condition, they will present the matter before the legislature in its proper light, and the disgraceful law will be so amended that it will not apply to educational institutions, and the charters already issued under it will be very promptly cancelled.

Some of the so-called dental colleges have no other existence than this State incorporation. They are owned and run by one man, and he, perhaps, sails under a false name. Of course, if they give no instruction whatever, and yet confer degrees, they are amenable to the law against fraud. But their diplomas are not offered at all in this country, being only advertised abroad. They know very well that if they attempt to ply their trade at home they will speedily be brought to grief, and so they permit no proofs of their work to come to light in America. There is no indication of their business at their published address, and any letters sent to them from this country are carefully left unanswered. Their work is done through European agents. We cannot locate them, and there are no proofs to be obtained in this country. Our *confrères* abroad complain bitterly of these swindlers, but they do not comprehend the situation, and when we ask them to obtain the proofs of their villany, they reply that the miserable affairs are under our immediate notice, and we should get the testimony here.

Sometimes our professional journals, and some of our prominent men, and even professional organizations, instituted for the purpose of regulating dental practice here, unwittingly further the objects of these men by falsely charging that respectable schools are practically engaged in the same business of granting irregular degrees, and thus they efface the line of distinction that the reputable colleges have been striving to set up. It is a singular fact that nearly or quite every application which approved colleges receive for irregular degrees comes from Europe; and because of these miserable villifications of respectable schools by American dentists acting with more zeal than discretion, and more fervor than knowledge, there is not an American college that is free from these insulting applications.

This is the condition that confronts us in America. This Association has done what it could, and advanced as fast as it could. It has been embarrassed by the lack of co-operation, and even by the active opposition, of those to whom it had a right to look for help. It has been denounced because it has not taken the radical steps demanded by men who have little comprehension of the difficulties to be met, and who do not understand that the tone of the colleges and the profession as a whole can only be advanced by a movement that is made as a whole. At the most critical moment the ground that had been gained has been lost through the absolute refusal of some of the colleges to vote to sustain the most moderate requirements, and it may almost be a matter for astonishment that so much has been accomplished. This Association has sharply drawn the line between the reputable and the disreputable schools, and despite the fact that over-zealous and unwise men have been industriously engaged in effacing it, and confusing the good with the bad by claiming that all have the same character, in this country the distinction is well known. It should be, and, if these ill-advised strictures are abandoned, it will soon be, as well comprehended abroad. All that is necessary is to scan the list of the members of the National Association of Dental Faculties, and if the name of an institution granting a diploma is not found in it, that document is unacknowledged by this Association. If any college that has a membership in this Association grants a degree or accepts a student irregularly, the faith and honor of every other member is pledged to inflict the most condign punishment upon presentation of the proofs.

But it has been charged that violations of the rules have been committed by members without subsequent punishment. There appears to be an impression that it is the duty of the Association to discipline a college upon mere rumors and to inflict punishment without proofs. This would be the rankest injustice. There has never yet been definite charges made against a college by any responsible party, with accompanying proofs or positive information where evidence could be found, without the most thorough investigation of the case. It has been charged before your committee that the case first cited was such a one. But in that case the implicated college corrected the error of its own volition. The remedy for infraction of our regulations thus rests in the hands of every respectable member of our profession; for so carefully has this Association guarded this point that it has appointed a committee with plenary powers for the express purpose of investigating charges of irregularity brought between the sessions, thus offering swift as well as exact justice.

As to the irregular colleges, your committee considers it the imperative duty of this body to employ every possible means for their exposure and suppression. We believe that it should protect the good name of American dentistry and American educational institutions. In this faith your committee, through its chairman, authorized the expenditure of a reasonable amount of money in the prosecution of investigations of unrecognized and irregular schools, and secured the co-operation of a thoroughly competent man for this work. As a consequence, considerable progress has been made in the unearthing of some of them. But it will probably take years of persistent effort to accomplish all that is desirable. We have received the most encouraging letters from our *confrères* in Europe, and have been materially aided by some of them. We have been assured that if such work is continued it must result in the higher appreciation of this Association in Europe, and in the perceptible raising of the estimation in which our degree is there held. Hence we feel warranted in urging upon you increased zeal in the prosecution of the work already commenced.

In view of all the considerations that have been presented in this report, your committee recommends the adoption of the following resolutions:

*Resolved*, That a standing committee of five be appointed each year by the president of this Association, to be called the Committee on Foreign Relations, whose duty it shall be to report each year upon the relative status of dentistry in America and Europe, and to suggest any measures that, in the opinion of its members, will promote the welfare of our common profession and the usefulness of the distinctive American dental degree.

*Resolved*, That the Committee on Foreign Relations be instructed to use its utmost diligence in ferreting out fraudulent or irregular colleges, and the granting of degrees irregularly by recognized colleges, should this be done, and to leave nothing undone within their power to bring to justice institutions granting irregular degrees or degrees irregularly. To this end this Association authorizes the committee to expend any reasonable sum of money, which, if necessary, shall be raised by some fair assessment of the colleges of this Association.

*Resolved*, That an Advisory Board, to consist of not more than three qualified persons from each of the following-named countries of Europe, be appointed by this Association, to the member or members of which the papers of any foreign applicant for matriculation in any American dental college shall be referred for verification or endorsement, it being understood that such papers shall be referred to the member or members of the board appointed for the country of which the applicant is or has last been a resident. The countries to be represented shall be—1, Great Britain; 2, Holland and Belgium; 3, Denmark, Norway, and Sweden; 4, Russia; 5, Germany; 6, Austria and Hungary; 7, Italy and Greece; 8, France; 9, Spain and Portugal; 10, Switzerland and Turkey.

W. C. Barrett,

S. H. Guilford,

D. J. McMillan,

F. D. Weisse,

A. H. Fuller,

*Committee.*

The resolutions were unanimously adopted, and the committee continued as "The Standing Committee on Foreign Relations," J. D. Paterson being appointed in place of D. J. McMillan, elected president of the Association.

The committee was authorized to appoint the Foreign Advisory Board.

It earnestly invites the co-operation of American dentists at home and abroad. Letters should be addressed to W. C. Barrett, chairman, 208 Franklin Street, Buffalo, N. Y., U. S. A.

